# Enhancing Single‐Domain Antibody‐Based Protein Degradation for Synucleinopathies

**DOI:** 10.1002/alz.090359

**Published:** 2025-01-03

**Authors:** Yixiang Jiang

**Affiliations:** ^1^ New York University Grossman School of Medicine, New York, NY USA

## Abstract

**Background:**

Synucleinopathies lack cures. Antibody therapies targeting α‐synuclein aim to inhibit aggregation and enhance degradation, but have limited brain entry because of size (150kDa). Smaller single‐domain antibodies (sdAbs, 15kDa) have substantially improved brain uptake. Previously, we developed sdAb‐based probes (2D10 and 2D8) enabling specific α‐synuclein imaging in mice post i.v. injection, correlating with lesion burden, indicating therapeutic and diagnostic potential (Jiang Y et al Sci Adv 2023).

**Method:**

To enhance sdAbs’ efficacy, we developed 2D8‐PEGn‐T (n = 2,4,6), linking 2D8 sdAb and thalidomide (T) with different PEG linkers to promote proteasomal degradation (sdAb‐PROTAC). We assessed sdAb binding affinity to α‐synuclein preparations by biolayer interferometry, examined α‐synuclein degradation in M83‐mouse primary culture, and evaluated therapeutic efficacy in M83‐synucleinopathy mouse model (n = 17). Using 2D10 sdAb linked to a near‐infrared‐tag and In‐Vivo‐Imaging‐System, we assessed brain α‐synuclein burden pre‐treatment. Mice with similar burden received 3 i.v. sdAb injections (molar equivalent: 100 µg of 2D8) 3 days apart, with re‐imaging after 3 days, followed by brain extraction for analyses of α‐synuclein clearance and potential toxicity.

**Result:**

2D8‐PEGn‐T’s affinity after conjugation was comparable to 2D8’s affinity. 2D8‐PEG4‐T targeted α‐synuclein and Cereblon, inducing ubiquitination and proteasomal degradation. In the culture, 2D8‐PEG4‐T prevented α‐synuclein‐induced toxicity and reduced α‐synuclein levels via lysosomal‐ and proteasomal‐pathways, outperforming unmodified 2D8 sdAb that mainly cleared α‐synuclein via the lysosomal‐pathway. In the M83‐synucleinopathy mouse model, 2D8‐PEG4‐T significantly (81%, p = 0.0049) reduced α‐synuclein brain signal, surpassing 32% for 2D8 (p = 0.3195) vs PBS control. Western blots showed 2D8‐PEG4‐T to be more efficacious (89‐93% reduction, p<0.0001) in reducing insoluble total and pS129 α‐synuclein, compared to 2D8 (59‐69% reduction, p = 0.0015 and 0.0016) vs PBS control. Additionally, 2D8‐PEG4‐T decreased soluble total and pS129 α‐synuclein levels by 70% (p = 0.0072) and 90% (p = 0.0001), surpassing 2D8 that did not significantly reduce soluble total (p = 0.8643) or pS129 α‐synuclein (reduced by 34%, p = 0.1018), compared to PBS control.

**Conclusion:**

2D8‐PEG4‐T enhances α‐synuclein proteasomal degradation while maintaining 2D8’s lysosomal clearance. Its superior α‐synuclein clearance, compared to unmodified 2D8 sdAb in vitro and in vivo, highlights its therapeutic potential for synucleinopathies. Small sdAbs with improved brain entry and potency may enhance clinical benefits in antibody‐based therapies.

